# Implementation of the remote measuring system for addiction patients in rehabilitation applying vital sensor

**DOI:** 10.1080/13102818.2014.949049

**Published:** 2014-10-28

**Authors:** Myung-Jae Lim, Ki-Young Lee, Young-Man Kwon

**Affiliations:** ^a^ Department of Medical IT and Marketing, Eulji University, Seongnam, Korea

**Keywords:** addiction patient, u-Healthcare system, vital sign, ubiquitous network

## Abstract

Recently, with the rapid development of related ubiquitous industries, ubiquitous-Zone (u-Zone) development is being promoted to build a ubiquitous environment within a specific area. From a health care system perspective, in particular, u-Zone is expected to contribute to reducing cost and effort to manage patients’ condition such as in-patients, addiction patients and mental patients. In contrast, the current health care system only targets specific persons or continues to expand the internal system of hospitals. As addiction patients are on the rise in terms of drug addiction, including alcohol and narcotics, behavioural addiction attributable to the exposure to games, gambling, Internet and mobile communications and shopping is also becoming a problem. That is why it is difficult to collect data for the daily addiction status, which causes difficulties in systematic management and accurate diagnosis. Therefore, this paper suggests a remote measuring system to collect continuous condition data, which monitors the addiction patients via the vital sign measuring sensor within u-Zone. That is, the system collects their condition information from the sensors measuring heart rate, body temperature and acceleration, based on which the specialists determine the patient's emotional state. These data are expected to become the basis of diagnosing and managing addiction patients.

## Introduction

Modern society is jumping into a ubiquitous era amid the efforts to develop information technology (IT) and improving network environment.[1] Recently, with the emergence and rapid development of businesses related to ubiquitous networks, today in Korea different public institutions are pursuing such relevant projects to provide local-level ubiquitous network service.[2]

Social stress contributes allegedly to the increasing occupancy rate of the patients addicted to drugs, including alcohol and narcotics, among mental patients. Such addiction patients change their emotions in a wider range even within a short period of time; for effective treatment, their daily routine data should be collected in a large volume and tracked to see the cycle of emotional change. However, since the existing system only targets persons or the hospitals mainly utilize high-budget equipment, it seems difficult to extract data from the ordinary lives of specific patients.

Thus, this paper suggests a remote monitoring system to more accurately collect the patients’ condition data while they lead their daily life, by means of the sensors attached to addiction patients to read their heart rate, body temperature and acceleration within ubiquitous-Zone (u-Zone). The sensors exchange data in a ZigBee communication method between sensor nodes to send such collected data via a gateway server to the hospital where specialists conduct.[2,3]

## Materials and methods

### Ubiquitous network

A ubiquitous network not only satisfies the needs of self-generation/management, but also adapts to a variety of communication environments and services, thereby providing efficient network infrastructure. To this end, ubiquitous networks are designed to have a hybrid mesh network where a network can be composed by itself with expansibility, supporting specific element technology to meet the needs as a ubiquitous network.[4]

**Figure 1.**
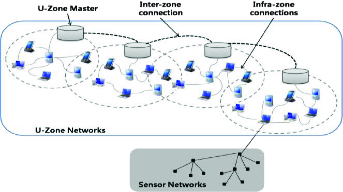
u-Zone network architecture.

A ubiquitous network is composed of a ubiquitous space, the zone network around the u-Zone master, while it is a hybrid mesh network consisting of ad hoc networks between terminals within the domain. Figure 1 shows the ubiquitous network architecture. The upper u-Zone master is an immovable compact device, less restrained by resources compared to general terminals and easy to install, working based on a ZigBee interface that links the multiplex WIFI interface and the sensor network.

### Bluetooth, ZigBee

The technology according to the standard IEEE 802.15.4 (Institute of Electrical and Electronics Engineers) for ZigBee communication – a wireless local area network – is a new advanced technology one step ahead of the existing radio-frequency communication technology (Table 1).[5]

**Table 1.** Comparison of Bluetooth and ZigBee.

Market name	Bluetooth	ZigBee
Standard	802.15.1	802.15.4
Application	Cable replacement	Monitoring and control
System resources	250 KB	4–32 KB
Battery life (days)	1–7	100–1000 +
Network size	7	255
Bandwidth (kbps)	720	20–250
Transmission range (m)	1–10 +	1–100+
Success metrics	Cost, convenience	Reliability, power, cost

ZigBee communication technology has its highest bandwidth up to 250 Kbit/s, which is not enough to send and receive high-capacity information. However, this wireless Internet device is only fed with 50 mW, a sixth of any other device of its kind consuming electricity not less than 200 mW. As such, the wireless system has its battery life last longer.[6]

### u-Healthcare system

u-Healthcare is an area of technology that uses a large number of environmental and patient sensors and actuators to monitor and improve patients’ physical and mental conditions in a networked system. u-Healthcare system is a system with ubiquitous access to e-health. The service of u-Healthcare system is not restricted only to clinics and hospitals but is available at every place. u-Healthcare system is about management of EMR (Electronic Medical Record), PACS (Picture Archiving Communication System), HIS (Hospital Information System), OCS (Order Communication System) and enhancing health care by increasing the availability of patient monitoring devices, and various health data of the patients to physicians.[7]

### Addiction types and symptoms: brief review

Addiction is largely divided into two types: psychological dependence and physical behaviours.[8] Psychological dependence refers to resorting to drug due to pressure or stress felt in society, which usually includes addiction to alcohol, nicotine and drugs. Stopping the consistent dose causes anxiety and nervousness, leading to so-called withdrawal symptoms such as hand tremor.[9] Those addicted should be subject to longer continuous treatment and even after treatment, they may feel difficulty in adapting to society and are most likely to relapse.[10]

Addiction to physical behaviours showing certain symptoms is divided into intangible and tangible behavioural addictions: for instance, intangible ones include addiction to relationship, anger, dating and religion; and tangible ones include addiction to work, gambling, computer or mobile game, sex, shopping and exercise.[11,12] In this paper, we tested the change of state about participant's body temperature, pulse and acceleration after computer or mobile game. We anticipate it to be able to make a distinction between the general group and the high-risk one.

### Hardware specification

**Figure 2.**
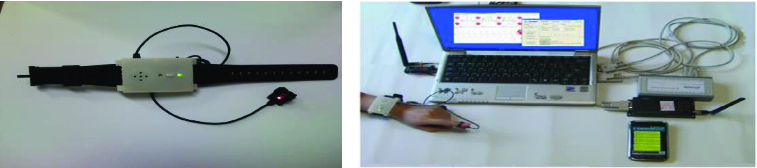
PPG signals obtained using pulse-measuring equipment.

The proposed technique used the following hardware specification in its performance evaluation: AMD Turion(tm) X2 Ultra Dual-Core Mobile ZM-85 2.30 GHz, 2 GB RAM and the O/S system of Microsoft Windows XP SP3. Figure 2 shows a portable wireless pulse-measuring instrument developed through this paper, which can obtain a slight pulse wave from the finger part, using a photo-plethysmography (PPG) sensor. The configuration is made to detect the light emitted from the light source using LED and CDS Cell by an optical sensor after being reflected in the living tissues, including blood vessels.

**Figure 3.**
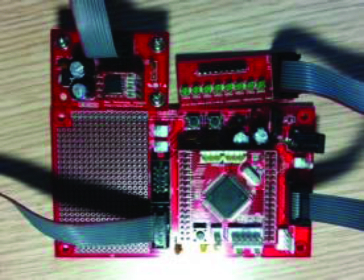
Acceleration sensor.

Figure 3 shows the acceleration sensor used in this paper (FREESCALE's A7260). The microprocessor sensor module used is ATMEL's Atmega128.

### Subjects

The subjects who took part in the study included 12 high-risk group patients and 12 general group patients (non-addicts). All participants were volunteers. An especially high-risk group are those suffering from game addiction. As they had scored high on the diagnostic scale, they had been enrolled in a game addiction counselling programme operating at the Eulji University's addiction rehabilitation centre.

## Results and discussion

### System flow chart

**Figure 4.**
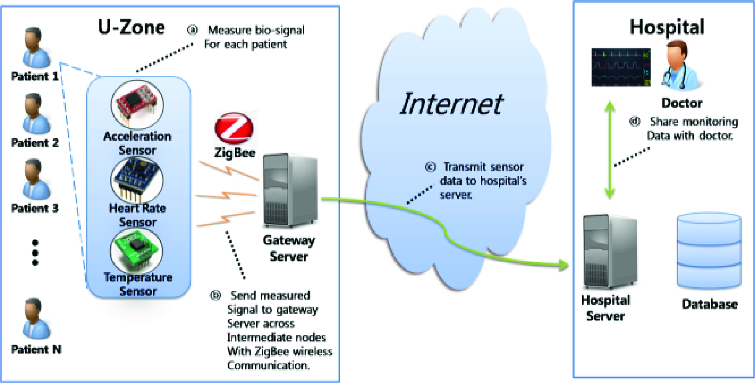
Health care procedures organized for addiction patients.

The proposed system is attached to the addiction patients who can live their ordinary lives, within u-Zone, by which their conditions are sent to their hospital and monitored by specialists in real-time (Figure 4).

Each sensor measuring acceleration, heart rate and temperature collects and sends data to the adjacent sensor, throughout which the ZigBee method is used. The acceleration sensor works using a triple-axis accelerometer that can measure in the directions of the *x*-, *y*- and *z*-axes. The heart rate sensor works based on pressure and the temperature sensor, using infrared rays. The gateway server in u-Zone sends the information from each sensor to the hospital server which stores such received information in its database and supports monitoring of the data by specialists.

**Figure 5.**
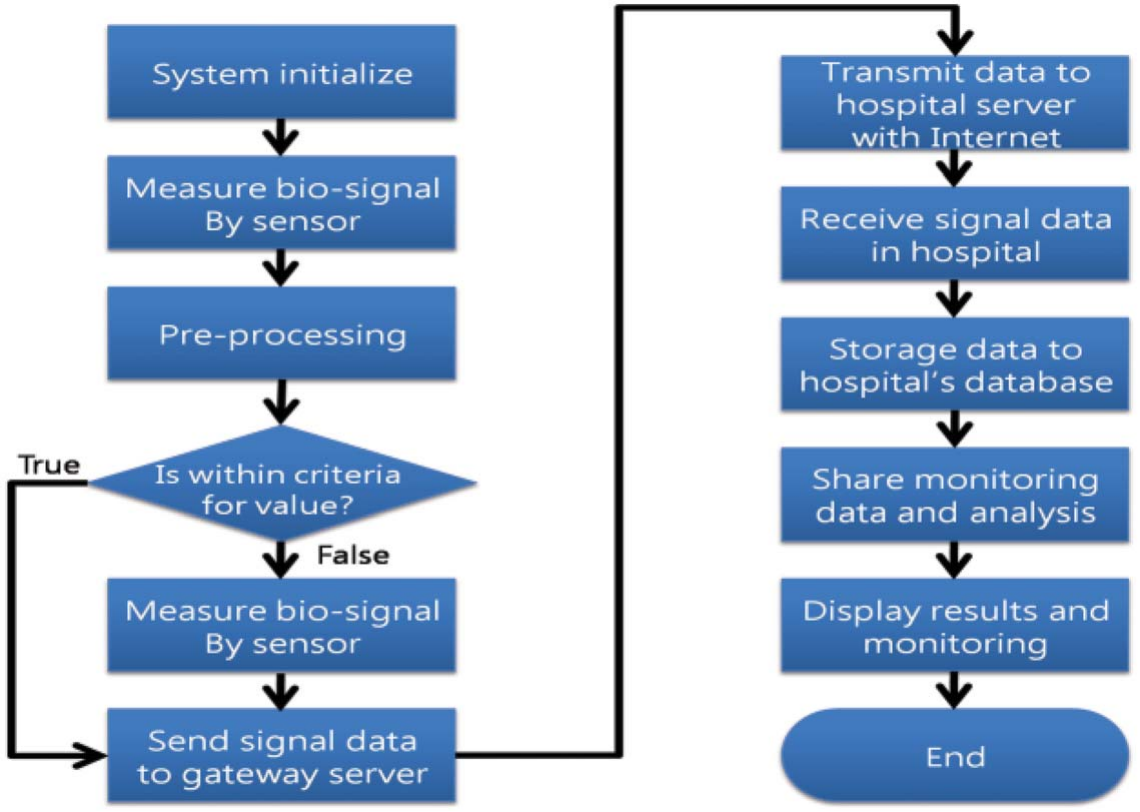
Flow chart of the bio-signal processing.

Figure 5 shows the entire flow of the system. First, the system is initiated and it measures vital signs via the sensors for acceleration, pressure and temperature attached to each patient. This information undergoes a pre-treatment process where unnecessary parts are excluded in order to simplify the complexity of calculation in the course of processing.

In addition, the reference values in Table 2 were referred to determine the level of each measurement. The acceleration on average was found to amount to 0.5–5.5 km/h, while the heart rate on average read 45–120 bpm and the body temperature on average was 35.5–37.5 °C. If the measured value of acceleration, heart rate and/or temperature deviates from the reference scope, the value is adjusted to fall within the references. For example, when a measurement goes above the reference value range, the measurement is designated as the upper reference value, whereas if a measurement is below the reference range, the measurement is designated as the lower reference value.

**Table 2.** Status-test standard.

	Avg. accelerator (km/hr)	Heart rate (bpm)	Temperature (°C)
5	5.5	120	37.5
4	5.0	110	37.3
3	4.5	100	37.1
2	4.0	90	36.9
1	3.5	80	36.7
0	3.0	70	36.5
−1	2.5	65	36.3
−2	2.0	60	36.1
−3	1.5	55	35.9
−4	1.0	50	35.7
−5	0.5	45	35.5

The level classifying each measurement is a figure to track the emotional change on an 11-point scale between −5 and 5. The mean from the three levels under which the relevant measurements fall is obtained in order to calculate the comprehensive level value: if this level value is 0, the patient is in an ordinary emotional state; the closer to −5 the value is, the more the patient feels depressed (depression) and the closer to 5 the value is, the more the patient becomes excited (anger, agitation).

The vital signal information of patients adjusted to the figures within the reference scope in such a way is sent to the gateway server in u-Zone via medium nodes. The server sends this information to the hospital server via the Internet and the hospital server stores the received information in its database. In this way, one can check both the data measured via each sensor and the results of the patients’ emotional level evaluated in a final stage, for which one can continue to monitor.

### System architecture model

**Figure 6.**
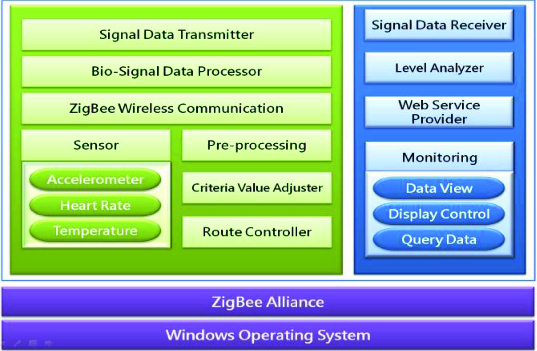
System architecture based on sensor.

Figure 6 shows the entire system structure and the detailed architecture model of each module. This system uses Microsoft Windows as an O/S system and works based on ZigBee Alliance environment. The vital signs measures via those sensors are processed under Bio-signal Processor, for which the pre-treatment is performed basically. Furthermore, a Criteria Value Adjuster plays a role in adjusting measurements from each sensor so as not to deviate from the reference scope.

Signal Data Transmitter serves as a sender of this signal information, while the information among sensors is transmitted via a ZigBee Wireless Communication module on a wireless basis. The information from each sensor is repeatedly sent to the adjacent sensor node, which finally arrives at the gateway server. Moreover, all routes in the course of transmission are systematically controlled by a Route Controller. The monitoring module mostly serves as a data viewer to display the measurements of acceleration, heart rate and temperature as well as measurement information calculated by the Level Analyzer. This module also works as a display controller to rearrange information at a certain interval and as a query data controller to filter information in accordance with the desired condition and request.

The Web Service Provider module provides the web service where one can access and monitor the information, for which a proper authentication is required to prevent any security problems in advance.

### System implementation

#### Algorithm for managing addiction patients

**Figure 7.**
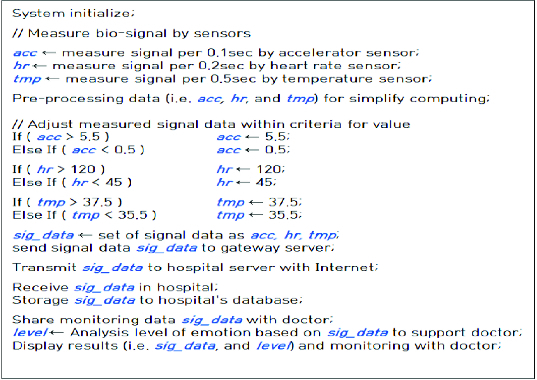
Status information management algorithm.

Based on the information from the sensors attached on addiction patients, an algorithm works for monitoring and management as shown in Figure 7.

First, the entire system is initiated and then a vital sign measured by each sensor is allocated to the relevant variable. As shown in Figure 7, *acc* is a signal value measured every 0.1 seconds by an accelerometer, *hr* is a signal measured every 0.2 seconds by a heart rate sensor and *tmp* is a signal measured every 0.5 seconds by a temperature sensor. The data measured by the accelerometer are used to recognize the patient's movement.

The practical analysis conducted by the algorithm performs the pre-treatment for normalization and optimization in terms of the data such as *acc*, *hr* and *tmp* in order to simplify the processing and minimize the calculation volume as follows:

1. Process missing data to make up for the information loss by using mean values.
2. Process the noise generated during transmission via a low-pass filter.
3. Process noise data to eliminate modified value.
4. Integrate and normalize various forms of data into a consistent unified data structure.

After pre-treatment, the estimation of the emotional level requires adjustment of each signal value not to deviate from the reference value scope. If any signal is outside the scope, *acc* should be adjusted to amount to the value between 0.5 and 5.5; *hr* between 45 and 120; and *tmp* between 35.5 and 37.5.

### System interface

**Figure 8.**
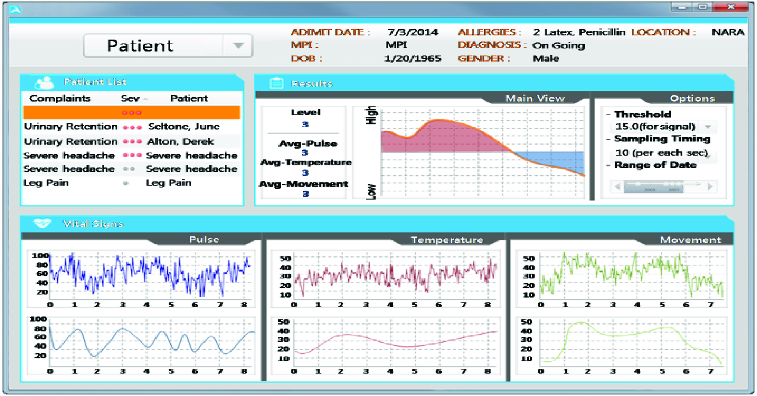
Status information check view.

The monitoring screen – the major interface in the proposed system Figure 8 – displays identification information on the upper screen, e.g. name, address and gender of the patient currently clicked; on the left screen, the list of patients to whom the sensors are attached in u-Zone; and on the bottom screen, the measurements by the sensors of pulse, temperature and acceleration.

The measurements are referred to the following:

1. Systolic/diastolic blood pressure (BP) of 120/80 as a reference value.
2. Heart rate (HR) during interphase of 6085 min^–1^ as reference.
3. Respiratory rate (RR) of 12–18 min^–1^ as reference.
4. Body temperature (BT) of 35.5–37.5 °C as reference since the steady-state deviation may become larger due to various factors including environment and measurement period (Table 3).

**Table 3.** Temperature sensor measurement criteria by body part.

Body part	Measuring period	Advantages and shortcomings
Oral cavity (37 °C)	3–5 min	Easy to measure; contraindicated after eating, smoking or oral surgery, or in the case of stomatitis, or in infants or unconscious patients
Eardrum (37 °C)	1–2 s	Quick to measure; may largely depend on external effects; difficult to be inserted in the case of newborn babies and infants due to the external auditory meatus structure
Rectum (37.5 °C)	2–3 min	More reliable alternative in the case of difficult/impossible measurement of oral temperature; may cause patients to experience embarrassment or anxiety
Armpit (36.4 °C)	6–10 min	Safe; lowest cost; usable for infants or unconscious patients; longer time needed form measurement and requirement for a nurse to help maintain the same position

### Experimental results

The results of the experiments for the 12 above-mentioned high-risk group patients and 12 general group subjects include three parameters: pulse, body temperature and acceleration separately. Each value is an average of 10 tests. Each parameter is individually measured before and after a condition.

**Figure 9.**
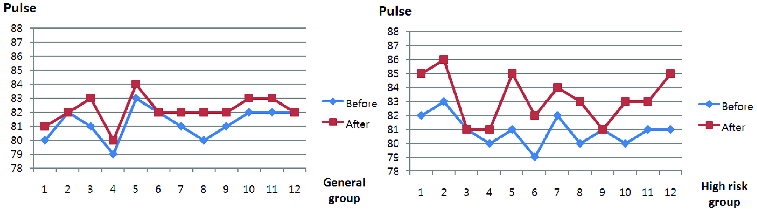
Pulse measurements (bpm).

In the condition labelled as ‘before’, the subject attached the sensor to their body and behaved as normal generally within the u-Zone. In the condition labelled as ‘after’, they attached the sensor and played a game in the u-Zone. Figure 9 shows the pulse measurement values. The deviation in the general group between condition ‘before’ (81.25 bpm on average) and ‘after’ (82.16 bpm on average) was about 0.91 bpm, whereas in the high-risk group the deviation between condition ‘before’ (80.91 bpm on average) and ‘after’ (83.25 bpm on average) was about 2.33.

**Figure 10.**
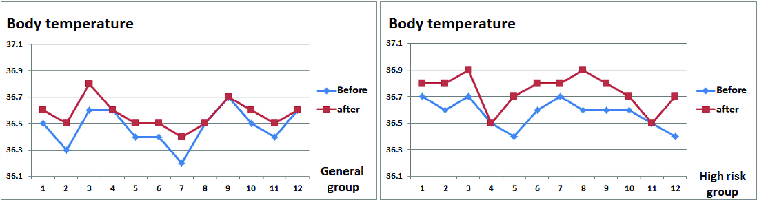
Body temperature measurements (°C).

Figure 10 shows the body temperature values in the two groups. In the general group, the deviation was about 0.09 °C (an average of 36.47 °C ‘before’ and 36.56 °C ‘after’) vs. 0.17 °C in the high-risk group (an average of 36.57 °C before and 36.74 °C after).

**Figure 11.**
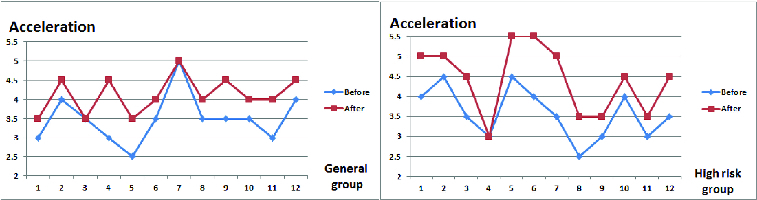
Acceleration measurements.

The acceleration measurement values are shown in Figure 11. The deviation in the general group was about 1.3 between condition ‘before’ (86.0 on average) and ‘after’ (87.3 on average), while in the high-risk group it was about 1.67 (on average 86.16 ‘before’ and 87.3 ‘after’).

As a whole, the high-risk group's deviation was higher than that in the general group in the case of all measurements. Consequently, the results from this experiment indicate that the system can distinguish between high-risk groups and others. This suggests that it is a practical tool for remote measurement of addiction patients.

## Conclusions

This paper proposes a network-based remote care system for addiction patients, by which the patients are monitored and managed continuously based on their condition measurements. The proposed method is to attach sensors measuring acceleration, heart rate and temperature, in such a way that each sensor transmits the received data to the adjacent sensor. The inter-sensor communication works by the ZigBee method. The gateway server in the u-Zone sends the data from each sensor to the hospital server, which stores the data in a database for specialist monitoring. In this way the system ensures a systematic management of patients and performs real-time monitoring; even when an emergency occurs, the system is expected to respond swiftly. Further studies will be aimed at complementation of measured data and improvement of the recognition accuracy by introducing additional sensors to the proposed system.
